# Determinants of adolescent childbearing in Ethiopia, analysis of 2016 Ethiopian demographic and health survey: a case-control study

**DOI:** 10.4314/ahs.v22i2.54

**Published:** 2022-06

**Authors:** Mengie Zinie Abita, Mamo Desalegn Girma, Tegegne Alemnew Wale

**Affiliations:** Mizan-Tepi University, Midwifery

**Keywords:** Teenage, childbearing, health, sexual, demographic

## Abstract

**Background:**

Pregnancy and birth complications experienced by adolescents are also problems of older women. But it is severe among the young due to physical immaturity and social condemnation from basic reproductive health services. The study was aimed to analyze determinants of adolescent childbearing in Ethiopia using the Ethiopian demographic and health survey.

**Method:**

The data source for this study was the 2016 demographic and health survey. Records of 359 cases and 1436 randomly selected controls (1:4 ratio) were included in the analysis. Adolescent childbearing was the main outcome variable and the independent variables were sociodemographic and sexual & reproductive factors. Multivariable logistic regression analysis was used to identify factors associated with adolescent childbearing.

**Result:**

The mean age of girls at first cohabitation was 15.28 ±1.64 and the mean age of first birth was 16.47±1.35. Adolescent childbearing was found to be higher in the Afar region (34.8%), and the lowest was in Addis Ababa city (4.1%). Finding from the multivariable analysis showed that place of residence, survey time age, and age at first sexual intercourse were the factors that have an association with adolescent childbearing. The odd of childbearing was higher among rural residents (AOR = 1.74; 95 % CI: 1.12, 2.72), early (<18 years) initiation of sexual intercourse (AOR =12.5; 95% CI: 5.97,25.18) and the risk is also higher among older teenagers (AOR =7.92; CI:3.92,15.90).

**Conclusion:**

Place of residents, age, and timing of first sexual intercourse was found to be the influencing factors of adolescent childbearing. Our finding indicates that the place of residence of the adolescent mothers must be considered in planning policies that attempt to disrupt successive cycles of socioeconomic deprivation. Public health interventions should focus their programs to be based on community and aim on prevention of early sexual intercourse and marriage.

## Introduction

Adolescence is a period of vulnerability in human development as it represents a transition from childhood to physical and psychological maturity.it is in this period that they learn and develop skills on critical aspects of their health and their body got matured. Childbearing during adolescence is not only a risk factor for adverse birth outcomes, but also harms the future well-being of the mother and the child^1^. Due to lack of adequate sexual and reproductive health (SRH) services adolescents are mostly exposed to early and unprotected sexual intercourse, unintended pregnancy, unsafe abortion, HIV infection, substance abuse, child marriage, and other SRH problems^2^.

Poor maternal conditions are the leading cause of mortality among girls aged 15–19 globally. In 2018, the estimated adolescent birth rate worldwide was 44 births per 1,000 girls aged 15 to 19, and in West and Central Africa, it was 115 births, the highest regional rate in the world. In developing regions every year, an estimated 21 million adolescent girls aged 15–19 years become pregnant and around 12 million of them give birth and at least 777,000 births occur to adolescent girls younger than 15 years in this region^3^,^4^.

Adolescent pregnancy is a major reproductive health challenge problem in Ethiopia. The 2016 Ethiopia Demographic and Health Survey (EDHS) report showed adolescent pregnancy rates of 13% 5. A Study in Ethiopia shows a slow decrement in adolescent childbearing rate from 2000 to 2016(16.5% to 12.5%) 6. According to the 2013 UNFPA report, Ethiopia was ranked among the top 10 countries with the highest number of women aged 20 to 24 years old and who gave birth by their eighteenth birthday^7^.

Adolescent pregnancy and childbearing have multidimensional implications for a nation such as educational opportunity, population growth, and ill-health of women and children. As a result, prevention of early marriage and reduction of adolescent pregnancy has been the focus of attention by several governmental and non-governmental organizations^8^. Previous studies have shown that maternal and perinatal morbidity and mortality can be reduced by lowering the high rate of teenage pregnancy in developing countries^9^. Consequently, reducing the high rate of adolescent pregnancy and maternal mortality is considered as the key Sustainable Development Goals (SDG).

However, studies are limited at a national level that identifies the determinant factor for this higher burden of adolescent childbearing in Ethiopia. Therefore, this study is intended to investigate factors associated with adolescent childbirth using 2016 EDHS data.

## Methods

### Study setting

The world bank data about the Ethiopian population have evidence that it is the second-most populous country in Africa, with a population of 105 million in 201710. According to the report from 2016 EDHS, almost half (47%) of the Ethiopian population are found in the age group of < 15 years^5^. Administratively Ethiopia is divided into nine regional states (Tigray, Afar, Amhara, Oromiya, Somali, Benishangul-Gumuz, Southern Nations Nationalities and People (SNNPR), Gambela, and Harari and, two city administrates (Addis Ababa and Dire Dawa).

### Data set and population

The study used data from the Ethiopian demographic and health survey conducted in 2016, which is the fourth comprehensive survey. It was a community-based cross-sectional survey conducted from January 18, 2016, to June 27, 2016, across the country and it is available from the MEASURE DHS database at https://dhsprogram.com/data/available-datasets.cfm In this survey data was collected on household characteristics among women aged 15–49. For our study data were extracted from the women's questionnaire, particularly those of adolescents aged 15–19 years.

### Eligibility criteria

#### Inclusion criteria

**Cases:** We have included all adolescent girls aged 15–19 and gave birth

**Control:** Adolescents aged 15- 19 and who didn't give birth.

#### Exclusion criteria

Women who lack critical information were excluded from the analysis.

### Sample size and sampling procedure

The EDHS survey was designed to represent all regions and administrative cities found in the country. The 2016 EDHS survey participants were selected in two stages. Initially, a total of 645 enumeration areas (202 in urban and 443 in rural) were randomly selected proportional to the household size from the sampling strata, and in the second stage, 28 households per cluster were selected using systematic random sampling. A total of 18,008 sample households were selected and from this 16, 650 households (98% response rate) were successfully interviewed in 2016 EDHS. There were 16583 eligible women and 15683 women had been interviewed and 3498 adolescents have participated. For our analysis, we used 359 cases (adolescents aged 15–19 and gave birth) 1436 randomly selected controls among adolescents aged 15–19 and didn't give birth at the time of survey by considering a 1:4 case to control ratio.

### Study variables

The main outcome variable for this study was having birth during the adolescence period. It was ascertained by asking the age of women at the time of their first birth which is recorded as one variable in the survey data.

### Independent variables

The predictor variables were examined by categorizing them into the socio-demographic background and proximate determinants of teenage childbearing. The socio-demographic factor included the place of residence (urban and rural), the region (Tigray, Afar, Amhara, Oromia, Somalia, Benishangul, SNNPR, Gambela, Harari, Diredawa, Addis Ababa), sex of head of a household, household wealth (poorest, poorer, middle, richer, richest) and educational status of both women and husband (no education, primary, secondary, higher). The proximal factor included decision-maker for using contraception, age at first cohabitation, unmet need for contraception, knowledge of ovulatory cycle, age at first sex, contraceptive use, and decision-maker to marry. Knowledge of ovulation was measured based on their response regarding the timing of ovulation. Those who responded, “ at the middle” and “after menstrual bleeding” were considered as knowledgeable.

### Data processing and analysis

The data was obtained from the MEASURE DHS database and needed variables were extracted into a new SPSS file with appropriate modification of data form in a suitable fashion for our analysis. Summary of descriptive statistics was done for both cases and controls.

Simple logistic regression analysis was done first to identify those factors that show association with the outcome variable (giving birth during adolescence period) at the bivariate level. Then factors in the simple logistic regression analysis which have a P-value <0.25 were a candidate for multivariable logistic regression analysis. Finally, variables with a P-value <0.05 in the multivariable analysis were declared as they have a statistically significant association with the outcome variable with a 95 % confidence interval of the odds ratio. Final model fitness was checked by Hosmer-Lemeshow goodness-of-fit test with P > 0.05 which was 0.918.

## Results

### Socio-demographic characteristics

A total of 1795 women aged 15–19 (359 have at least one birth and 1436 who didn't give birth before) were included in the analysis. The mean age of adolescents was 17.06 ±1.36 years. About two-thirds of adolescents included were urban residents. Nearly 19% of adolescents were uneducated and more than two-thirds (68.6 %) were unmarried. Among those who are married (31.4 %) around 44 % of them were attending school before marriage. Approximately 37 % of adolescents married to those who didn't read and write. (table 1)

**Table 1:** Socio-demographic characteristics of adolescent girls in Ethiopia using the 2016 DHS data

Variables	Categories	Childbearing status	
Age		Cases(%)	Controls (%)
	<18 year	74(7.5)	911(92.5)
	≥18 year	285(79.4)	525(36.6)
Marital status	Never in union	14(1.1)	1218(98.9)
	Married	295(62.8)	175(37.2)
	Living with partner	11(61.1)	7(38.9)
	Widowed	1(33.3)	2(66.7)
	Divorced	32(53.3)	28(46.7)
	Separated	6(50)	6(50)
Residency	Urban	53(8.6)	566(91.4)
	Rural	306(35.2)	870(64.8)
Religion	Orthodox	74(10.9)	602(89.1)
	Muslim	213(27)	575(73)
	Catholic	1(12.5)	7(87.5)
	Protestant	66(21.1)	247(78.9)
	Other	3(50)	3(50)
Educational status	No education	128(38)	209(62)
	Primary	192(18.5)	846(81.5)
	Secondary	37(10.4)	318(89.6)
	Higher	2(3.1)	63(96.9)
Wealth index	Poorest	153(35.2)	282(64.8)
	Poorer	59(26.3)	165(73.7)
	Middle	57(23.1)	190(76.9)
	Richer	31(13.9)	192(86.1)
	Richest	59(8.9)	607(91.1)
Husband educational status	No education	112(61.9)	69(38.1)
	Primary	118(65.9)	61(34.1)
	Secondary	49(62.8)	29(37.2)
	Higher	24(53.3)	21(46.7)
	Don't know	3(60)	2(40)

Sexual and reproductive characteristics of adolescents About one-third (31.4 %) of adolescents started sexual intercourse before they turn 18 years. The mean age of girls at first cohabitation was 15.28 ±1.64 and the mean age of first birth was 16.47±1.35. Around 4.1% of adolescents were pregnant during survey time. More than one-fourth of adolescents didn't know when ovulation could occur and only 17 % of them reported correctly the possible time it could happen. The mean duration of time from marriage to birth is 39.82 months (Table 2).

**Table 2:** Sexual and reproductive health history of adolescents in Ethiopia using the 2016 DHS

Variables	Categories	Childbearing status	
Early sexual intercourse (<18 yr.)		Cases(%)	Controls(%)
	Yes	349(61.8)	216(38.4)
	No	10(15.6)	54(84.4)
	Never had sex	0	1166(100)
Age at first cohabitation	< 18yr	332(65.6)	174(34.4)
	≥18	13(22.8)	44(77.2)
	Never cohabitated	14(1.1)	1228(98.9)
Knows sources of family panning	yes	167(16)	880(84)
	No	105(17.6)	491(82.4)
Contraception use and intention	Using modern method	85(57)	64(43)
	Using traditional method	2(66.7)	1(33.7)
	Non-user intended to use later	121(12.3)	865(81.7)
	Does not intended to use	151(23)	506(77)
Knowledge about ovulation	During period	15(19.7)	61(80.3)
	After period	119(33.4)	237(66.6)
	Middle of the cycle	56(17.7)	260(82.3)
	Before period begins	27(19.4)	112(80.6)
	At any time	78(17.8)	360(82.2)
	Don't know	64(13.6)	406(86.4)
Decision to marry	My self	167(64.2)	93(35.8)
	Parents	171(58.8)	120(41.2)
	Other family/relatives	4(57.1)	3(42.9)
	Unmarried	3(60)	2(40)
Beating is justified following refusal to have sex	Yes	148(28.9)	364(71.1)
	No	208(17.1)	1007(82.9)
	Don't know	3(4.4)	65(95.6)

More than one-third (36.6 %) of participants didn't have the intention to use any contraceptive methods and 55% are none-users but intended to use later. Almost 37 % of adolescents didn't know their source of family planning methods. The majority (73.5 %) of them have a joint decision on using contraceptive devices to achieve their desired family size. Above one-fourth, (28.5%) of participants responded that beating is justifiable following refusal to have sex with their husband.

### Determinants of adolescent childbearing

Household wealth index, educational status, place of residence, age at first cohabitation, and beating following refusal to have sex were included in the multivariable logistic regression. In the multivariable model place of residence and age of cohabitation showed significant association with adolescent childbearing. The odds of childbearing among adolescents who were rural residents was about 1.74 (AOR = 1.74; 95 % CI: 1.12, 2.72) times higher than those whose residence was in urban areas. Age during survey time was also the other predictor that showed association with. The odd of teenage childbearing was 12.5 (AOR =12.5; 95% CI: 5.97,25.18) times higher among older (≥18) teenagers. In comparison to adolescents who had started sexual intercourse ≥ 18 years, the estimated odds of childbearing was7.92(AOR =7.92; CI:3.92,15.90) times higher among those who had initiated sexual intercourse before eighteen (Table 3).

**Table 3:** Bivariate and multivariable analysis of factors associated with teenage childbearing using data from Ethiopian 2016 DHS

Variables	Categories	Child bearing status		COR(95% C.I)	AOR(95% C.I)
		
		Case (%)	Control (%)		
Educational	No education	128(32)	209(62)	19.3(4.64,80.12)	2.58(0.46,14.55)
status	Primary	192(18.5)	846(81.5)	7.15(1.73,29.47)	2.41(0.44,13.15)
	Secondary	37(10.4	318(89.6)	3.67(0.86.15.56)	2.75(0.49,15.43)
	Higher	2(3.1)	63(96.9)	1	1
Place of	Urban	53(8.6)	566(91.4)	1	1
residency	Rural	306(26)	870(74)	3.76(2.75,5.12)	2.34(1.56,3.51)*
Household	Poorest	153(35.2)	282(64.8)	5.58(4.01,7.78)	1.12(0.50,2.47)
Wealth index	Poorer	59(26.3)	165(73.7)	3.68(2.47,5.49)	0.96(0.40,2.27)
	Middle	57(23.1)	190(76.9)	3.09(2.07,4.60)	1.04(0.44,2.50)
	Richer	31(13.9)	192(86.1)	1.66(1.04,2.64)	0.64(0.27,1.54)
	Richest	59(8.9)	607(91.1)	1	1
Beating	No	208(17.1)	1007(82.9)	1	1
justified for	Yes	148(28.9)	364(71.1)	1.97(1.55,2.51)	1.19(0.83,1.71)
refusal to have sex	Don't know	3(4.4)	65(95.6)	0.22(0.07,0.72)	0.39(0.08,1.69)
Early sexual intercourse	Yes	349(61.8)	216(38.2)	8.73(4.35,17.50)	7.92(3.92,15.90)*
(<18yr)	No	10(14.6)	54(84.4)	1	1
Age	<18	74(7.5%)	911(92.5%)	1	1
	.18	285(35.2)	525(64.8)	6.68(5.06,8.82)	12.25(5.97,25.18)*

* p < 0.05, COR crud odds ratio, AOR adjusted odds ratio.

## Discussion

This study was conducted to identify determinants of teenage childbearing in Ethiopia using 2016 EDHS. Teenage childbearing varied based on different socio-demographic factors and it was higher (34.8%) in the Afar region and lower (4.1%) in Addis Ababa. This indicates that much effort is needed to narrow such a gap and lower this high prevalence of teenage childbearing in pastoral regions. The possible explanation for such variation among geographical regions could be socio-cultural differences towards early marriage and childbearing and, accessibility and utilization of sexual and reproductive health services.

Our study has found that place of residence has a significant association with adolescent childbearing. Teenagers who reside in rural societies are at higher risk of having birth before they turn nineteen compared to those who lived in urban. This is in line with a finding from a community-based case-control study in Uganda and another study in Ethiopia^10^, ^11^. This implicates that comprehensive sexual and reproductive health issues are not well addressed in rural areas as equally as in urban one. Living in rural areas may expose them to early marriage and to give birth early and might be followed by complications related to physiological and anatomical unpreparedness. The possible underlying reason for such tragedy is the lack of necessary reproductive health services particularly for adolescents who are usually neglected, groups. And the issue is mostly taboo in such societies to be discussed^12^.

The age of teenagers is also found to be the determinant of adolescent childbearing. Being an older teen increases the odds of adolescent childbearing in comparison with the younger. This is in line with other studies^13^, ^14^,^15^. This indicates that late teenagers had been wrongly perceived as an actual age to have safe pregnancy and childbearing. This could be explained by as age increases, the tendency of involvement in sexual activities is higher. This sexual orientation might be followed by marriage, pregnancy, and childbirth. But it is consequential for them as it is early and they are not physiologically matured to have a healthy conception.

Early initiation of sexual intercourse was another predictor of teenage childbearing. Adolescent girls who started sexual intercourse before 18-year-old are more likely to have a birth in their teens compared to those who initiated sexual intercourse after eighteen. The finding is consistent with another studies in Ethiopia and brazil^11^, ^16^. The implication here is, the sexual practice in the earlier time is more likely to be unsafe and unprotected and it inevitably followed by conception and childbirth. Having early sexual intercourse might increase the risk of early pregnancy whether intended or not due to lack of information, unavailability of and low utilization of SRH services by adolescents, and sexual abuse by their intimate partner. This situation could endanger their sexual and reproductive health not only from complications of pregnancy but also with associated sexually transmitted infections.

## Conclusion

Our analysis showed that rural places of residence, being older teenage and early initiation of sexual intercourse are the influencing factors for the occurrence of adolescent childbearing. This is suggestive to pay critical attention to adolescent girls habituated in rural parts of the nation and on sexual and reproductive health education for early adolescents. So a reduction of teenage childbearing could be achieved by increasing accessibility of friendly SRH services for those neglected segments of reproductive age groups of girls. Particularly those rural residents need due attention as they are threatened by underlying multidimensional socio-economic and cultural factors. And public health interventions should give great emphasis to such vulnerable groups of women population.

## Data Availability

The data used for this analysis are available in the Ethiopian statistical agency and ministry of health

## Abbreviations

EDHS: Ethiopian Demographic and Health Survey
SDG: Sustainable Development Goal
SNNPRS: South Nation Nationalities and Peoples Regional State
SRH: Sexual and Reproductive Health
UNFPA: United Nation's Population Fund
WHO: World Health Organization

## References

[1] WHO (2007). Adolescent pregnancy – Unmet needs and undone deeds: A Review of the Literature and Programmes.

[2] WHO (2017). Adolescent health.

[3] UNFPA (2015). GIRLHOOD, NOT MOTHERHOOD Preventing Adolescent Pregnancy.

[4] Darroch JE, Woog V, Bankole A, Ashford LS (2016). ADDING IT UP : Costs and Benefits of Meeting the Contraceptive Needs of Adolescents.

[5] Central Statistical Agency (CSA) Addiss Ababa Ethiopia and ICF (2017). Ethiopia Demographic and Health Survey 2016.

[6] Kassa (2019). Trends and determinants of teenage childbearing in Ethiopia : evidence from the 2000 to 2016 demographic and health surveys. Ital J Pediatr.

[7] Loaiza E LM (2013). Adolescent pregnancy: a review of the evidence.

[8] Westoff CF (2003). Trends in marriage and early childbearing in developing countries. DHS comparative report.

[9] Olausson O, Cnattingius S, Haglund B (2011). Teenage pregnancies and risk of late fetal death and infant mortality. Br J Obstet Gynaecol.

[10] The World Bank (2019). The World Bank in Ethiopia: Overview.

[11] Ochen (2019). Predictors of teenage pregnancy among girls aged 13 – 19 years in Uganda : a community based case-control study. BMC Pregnancy Childbirth.

[12] WHO WHO DISCUSSION PAPERS ON ADOLESCENCE.

[13] Tuyiragize R (2016). Predisposing factors associated with teenage pregnancy in Lake Victoria Islands and Mountain districts of Uganda CURRENT STATUS : POSTED.

[14] Kurth F, Be´lard S, Mombo-Ngoma G, Schuster K, Adegnika AA (2010). Adolescence As Risk Factor for Adverse Pregnancy Outcome in Central Africa – A Cross-Sectional Study. PLoS One.

[15] Manzi F, Ogwang J, Akankwatsa A, Wokali OC, Obba F (2018). Primary Health Care : Open Access Factors Associated with Teenage Pregnancy and its Effects in Kibuku. Prim Heal Care.

[16] Gigante DP (2004). Risk factors for childbearing during adolescence in a population-based birth cohort in southern Brazil. Rev Panam Salud Publica/Pan Am J Public Heal.

